# Music, Pleasure, and Meaning: The Hedonic and Eudaimonic Motivations for Music (HEMM) Scale

**DOI:** 10.3390/ijerph20065157

**Published:** 2023-03-15

**Authors:** Merrick Powell, Kirk N. Olsen, William Forde Thompson

**Affiliations:** 1 School of Psychological Sciences, Macquarie University, Macquarie Park, Sydney, NSW 2109, Australia; 2 Australian Institute of Health Innovation, Macquarie University, Macquarie Park, Sydney, NSW 2109, Australia; 3 Faculty of Society and Design, Bond University, Robina, QLD 4226, Australia

**Keywords:** music and emotion, motivation, eudaimonic motivation, hedonic motivation, music containing violent themes

## Abstract

Many people listen to music that conveys challenging emotions such as sadness and anger, despite the commonly assumed purpose of media being to elicit pleasure. We propose that eudaimonic motivation, the desire to engage with aesthetic experiences to be challenged and facilitate meaningful experiences, can explain why people listen to music containing such emotions. However, it is unknown whether music containing violent themes can facilitate such meaningful experiences. In this investigation, three studies were conducted to determine the implications of eudaimonic and hedonic (pleasure-seeking) motivations for fans of music with violent themes. In Study 1, we developed and tested a new scale and showed that fans exhibit high levels of both types of motivation. Study 2 further validated the new scale and provided evidence that the two types of motivations are associated with different affective outcomes. Study 3 revealed that fans of violently themed music exhibited higher levels of eudaimonic motivation and lower levels of hedonic motivation than fans of non-violently themed music. Taken together, the findings support the notion that fans of music with violent themes are driven to engage with this music to be challenged and to pursue meaning, as well as to experience pleasure. Implications for fans’ well-being and future applications of the new measure are discussed.

## 1. Introduction

A prominent question in media research is what motivates people to engage with violently themed media when negative emotions associated with violence are normally avoided [1,2,3]. Described as a ‘paradox’, deliberately engaging with media that conveys difficult emotions such as sadness, anger, and disgust seems to contradict the assumed function of media—enjoyment [4,5]. However, although enjoyment and positive experiences are primary motivators and outcomes of musical engagement [6,7], people also seek out music that is not merely enjoyable or pleasurable [8].

The present investigation focused on ‘violently themed music’, defined in this study as music containing lyrics that depict overt actions of violence to a person or group. Such music is commonly found in certain subgenres of extreme metal and rap music, with bands such as Cannibal Corpse having sold millions of records worldwide with album titles including “Kill”, “Evisceration Plague”, and “Torture” [9]. The sonic elements of extreme metal music are often described as intense and aggressive, containing distorted instrumentation, use of extremely low and high frequency ranges, and vocal delivery that is growled or screamed [10]. Violently themed rap music predominantly evokes violence through lyrics, although the accompanying music can sometimes reinforce the lyrical imagery (e.g., gunshot sounds).

Violently themed music has been shown to elicit predominantly positive affective responses from its fans, and passionate fandom of such music has been shown to contribute to fans’ psychological well-being, despite the overtly violent lyrical content [11,12]. However, when compared to passionate fans of non-violently themed classical music, fans of violently themed extreme metal and rap music reported significantly greater negative affective experiences such as anger and fear, and lower levels of enjoyment and positive affective experiences in response to their respective preferred genres [11]. Similarly, “sad” music, often characterised by minor mode, slow tempo, and legato articulation [13,14], evokes sadness as a predominant emotional response from its listeners, alongside various positive affective responses such as wonder and peacefulness [15].

Fans of music containing negatively valenced themes appear to seek a balance or ‘sweet spot’ between the experience of positive and negative affective responses when engaging with these kinds of music. Although experiences of depressive symptoms and maladaptive emotion regulation processes, such as cycles of rumination, may be responsible for a small proportion of negative affective responses [16,17], we propose that fans also seek out negatively valenced music to learn about, experience, and confront difficult emotional experiences. Such experiences are likely to be rewarding and gratifying, even when they do not elicit significant levels of enjoyment or other positive affective experiences [18].

This kind of motivation has been described by Oliver and Raney (2011) as eudaimonic motivation, where people seek out challenging, meaningful, and thought-provoking media [4]. In contrast, with hedonic motivation, people seek out media for pleasure or enjoyment purposes. There are few systematic investigations of these types of motivations for music listening. The present study sought to validate an adapted scale of hedonic and eudaimonic motivation to understand the presence of these two different motivation types across a wide range of music fans. Further, we investigated the relationships between listeners’ motivation types and their affective responses to music and how such motivations differ between fans of violently themed music and fans of non-violently themed music. 

### 1.1. Motivation for Music Listening

Why are people motivated to engage in activities such as listening to music? People are motivated to engage in activities based on the perception that they are pleasurable, valuable, absorbing, and fulfilling certain psychological needs and goals [19,20]. The motivations that people may have for listening to music are numerous and complex. Studies have investigated different specific motivations for listening to music and the functions that music serves for its fans, observing that relaxation, mood regulation, aesthetic entertainment and appreciation, memory evocation, and social connection are common motivations for listening to music, amongst many others [21,22,23]. Uses and Gratifications Theory suggests people are active agents in the selection of the media they seek out and engage with, based on their belief that it will fulfil specific needs [24,25]. However, in the context of fans of music containing violent themes, there has been little investigation into whether they are specifically motivated by pleasure and the enjoyment of the music, be it from the violent lyrics themselves or other enjoyable musical qualities, or whether it is based on fulfilling other, more complex eudaimonic needs.

### 1.2. Hedonic and Eudaimonic Motivations

Qualitative evidence suggests that people often listen to music to experience challenging emotions such as sadness, anger, or fear, as well as for enjoyment [26,27]. Rather than seeking only enjoyment or pleasure, listeners appear to seek out experiences through music that address all aspects of life and its various meanings and purposes. Eudaimonic motivation is defined as the desire to engage in activities or experiences that are challenging and thought-provoking, to find meaning and purpose, and to experience psychological growth [19]. This term was developed from Aristotle’s concept of eudaimonia, which describes living in pursuit of one’s best self [20]. On the other hand, hedonic motivation is defined as the desire to engage with media purely to experience pleasure and other positive experiences, or to avoid pain [4].

The terms hedonic and eudaimonic are often discussed in the context of well-being, with psychological well-being being conceptualised as the presence of both hedonic well-being (the experience of pleasure and avoidance of pain) [28] and eudaimonic well-being (experiences of personal development and purposefulness) [29]. Music has been shown to significantly contribute to fans’ hedonic and eudaimonic well-being, including violently themed extreme metal and rap genres [12]. The role of eudaimonic enjoyment in positive psychosocial experiences of music listening has been hypothesised, whereby eudaimonic enjoyment may facilitate self-reflection—a common function of music listening [30,31].

Study 1 sought to develop a means of understanding the presence of hedonic and eudaimonic motivations in music fans, as no specific measure has been developed and validated for this purpose. This measure will allow for a nuanced understanding of whether different listeners and fans of different genres exhibit different motivations. We predict that music fans across a range of genres generally possess high levels of both hedonic and eudaimonic motivation for engaging with music. However, as these different motivations may reflect the desire to satisfy different needs, different motivations are likely to be associated with different affective responses to music. Study 2 investigated this potential association.

### 1.3. Motivations and Affective Responses to Music

Research has investigated the influence of musical experiences on emotions, detailing how music can evoke a broad range of affective and physiological experiences in individuals, groups, and societies [8,22,32,33]. Musical experiences of pleasure can result in feelings of joy, empowerment, and transcendence [11], as well as the psychophysiological sensations of musical ‘chills’ and ‘thrills’ (for a discussion, see [32]). These strong emotional and physiological responses have been associated with increased blood flow to brain regions related to reward [34]. While personal preferences, musical features, and cultural and contextual factors influence the kinds of music that can evoke such intense pleasurable experiences [8,32], it is unsurprising that pursuing pleasure and enjoyment in musical experiences is a primary motivator for many listeners and fans.

As hedonic motivations reflect the desire to experience pleasure and positive experiences through engagement with media, we predicted that hedonic motivation would be prominent in music fans and that it would be associated with positive affective responses to music. In other research, hedonic motivations for film have been associated with the enjoyment of comedies and action movies, as well as experiences of being excited, entertained, humour, and amusement [4]. Some hedonically motivated individuals may be unable to experience positive outcomes in response to music, partly due to individual personality variables such as high levels of obsessive passion [10], high levels of psychopathology [16,31], or maladaptive emotion regulation processes [35]. This reflects an important caveat of the Uses and Gratifications Theory: there may not always be an alignment between gratifications sought and obtained due to individual differences and the inability of the media to satisfy the desired outcomes [36]. Nevertheless, it is expected that most listeners who report high levels of hedonic motivation for music listening in a non-clinical population would expect to experience positive affective experiences.

Furthermore, Study 2 predicted that eudaimonic motivation would be associated with mixed affective responses to music. While pleasure remains a dominant motivator and experiential outcome of music engagement, people listen to music to evoke a range of complex feelings and challenging experiences, such as reflecting upon themselves, their identity and values, or the emotions of others in different historical or sociopolitical contexts [8,11]. Music and other media can provide spaces to confront difficult challenges in a ‘safe’ environment through a different cognitive frame [37]. Further, strong experiences with music, such as experiences of musical thrills, are not only associated with positive emotions and may be associated with the intensity of emotions, rather than just emotions with positive valance [32].

As eudaimonic motivation reflects the desire to have challenging and thought-provoking experiences with media, those high in eudaimonic motivation will likely have more complex and less directly positive affective experiences. While eudaimonic motivations for other activities have still been associated with enjoyment, positive affect, and even life satisfaction [38,39], eudaimonic motivation has been associated with mixed emotional responses of happiness and sadness for fans describing their response to favourite films [4]. Mixed emotions were measured by participants recording their happiness and sadness scores in response to the film separately, with the lowest score of the two being taken to signify the extent of the presence of both emotions being experienced at one time. This approach, known as the minimum index, is a commonly used and favoured approach to measuring self-reported mixed emotional responses [40,41,42].

Further, eudaimonic motivation has been associated with descriptors such as compassionate, inspired, introspective, and contemplative, and not with descriptors of ‘fun’ affect, such as amusement or humour [4]. As music is commonly used to reflect upon oneself and their identity [10,43], eudaimonically motivated music fans may still experience adaptive outcomes, but these positive outcomes may not be captured through the lens of positive emotions or affect, but other more complex processes that lead to meaning making and self-reflection.

### 1.4. The Potential Role of Eudaimonic Motivation for Fans of Violently Themed Music

Eudaimonic motivation may be particularly prevalent in fans of music containing violent themes, as fans may seek to learn about challenging experiences through music that contains themes of torture, assault, and death. Intentionally grappling with truth and meaning may require confronting more difficult aspects of life. While this may be challenging and even painful at times, it may also be rewarding and gratifying. This may explain why fans of violently themed music report a more balanced experience of positive and negative affective responses to their favourite music than fans of non-violently themed music [10]. Fans may simultaneously experience joy, wonder, and empowerment, but also feel angry or tense when passionately engaged with violently themed music. Further, listeners may be able to engage with the music through an ‘art schema’, whereby they can experience typically negative emotions but in a positive or constructive manner [37].

It is important to note that we do not predict that these eudaimonic motivations or mixed affective experiences are limited exclusively to fans of violently themed music. People may seek out challenging information and experiences from many different genres of music, and such engagement may still result in the experience of a balance of positive and negative affective experiences. For example, fans of sad music commonly report feeling ‘moved’ in response to sad music, which is considered a mixed but largely positive affective response [44]. Hence, although we predict that music fans across a range of genres will possess high levels of both hedonic and eudaimonic motivation for engaging with music, we predict that fans of violently themed music may possess greater eudaimonic motivation compared to fans of non-violently themed music.

## 2. Aims

The aims of this study were (1) to validate a new measure of hedonic and eudaimonic motivations for listening to music, (2) to understand the relationship between these motivations and affective responses to music, and (3) to compare these motivations and experiences for self-reported fans of violently themed music and fans of non-violently themed music. Three studies were designed to address these questions. Study 1 adapted and validated a measure of motivations for music. Oliver and Raney’s (2011) scale of hedonic and eudaimonic motivations, originally designed for film viewers, was adapted to pertain to music to assess the presence and levels of each motivation in fans of music [4]. Study 2 further validated the measure and investigated the relationships between hedonic and eudaimonic motivations and fans’ affective responses to music. Finally, Study 3 investigated whether the presence of these two motivations differed between self-reported fans of violently themed music and fans of non-violently themed music.

## 3. Study 1: Developing the Hedonic and Eudaimonic Motivations for Music (HEMM) Scale

The first study aimed to validate a self-report scale to measure two different motivations for listening to music, hedonic and eudaimonic motivations, termed hereafter the Hedonic and Eudaimonic Motivations for Music (HEMM) scale. The development of the scale was based on a previous measure of hedonic and eudaimonic motivations for entertainment consumption in film [4] and was adapted to music. The scale was originally developed by assessing open-ended responses from fans of various films and then was validated over four experiments with large samples of participants. Results showed strong support for the factor structure of the measure and its construct validity. For example, eudaimonic motivation was associated with preferences for non-fiction and drama films, whereas hedonic motivation was associated with comedies. Discriminant validity was confirmed by the substantial improvement in fit between a 1-factor and 2-factor model, showing that the two latent variables assess unique constructs. Hence, it was determined that this scale would be a suitable basis for adaptation to a measure for music. Study 1 also sought to understand the magnitude of the presence of each motivation type in a sample of passionate music fans. We predicted that passionate fans of music would exhibit high levels of both hedonic and eudaimonic motivations.

### 3.1. Materials and Method

#### 3.1.1. Participants

A sample of 202 participants (96 males, 102 females, and 4 who identified as neither male nor female or chose not to respond), collected through the survey platform Prolific, were recruited for this study. Participants were required to be fans of music and could be fans of any genre. Participants’ mean age was 25.4 years old (*SD* = 6.8), and ages ranged from 18 to 54. Participants listened to an average of 24.6 h of music per week (*SD* = 18.9). Although the sample size was below the commonly desired sample size of 300 or greater for exploratory factor analysis [45], it was decided that 202 would be sufficient if following Guadagnoli and Velicer’s (1988) guidelines of factor loadings [46]. These guidelines suggest that factors are interpretable at this sample size if they have four or five loadings of 0.6 or above, which were observed in the analysis, and thus the sample size was deemed appropriate.

#### 3.1.2. Measures

Twelve items were adapted from the Oliver and Raney (2011) scale by adjusting references to film such that they referred to music listening [4]. One example item is “I like music that challenges my way of seeing the world”, which was adapted from “I like movies that challenge my way of seeing the world”. Two items in the hedonic subscale required greater rewording, as the language was based on laughter. Because laughter is a less common motivator for music engagement compared to film engagement, these items were reworded to reflect positivity or enjoyment as a motivator. One example is “As long as I am having a good time, I enjoy music that may be considered ‘basic’ or ‘shallow’”, which was adapted from “I like movies that may be considered ‘silly’ or ‘shallow’ if they can make me laugh and have a good time”. Items on the original scale were measured on a 7-point Likert scale, which was maintained as the measurement scale in our adapted version. The final wording for all adapted items is presented in Table 1.

Five items from the Passion Scale [47] were also included to identify whether participants were passionate fans of music. Participants were asked to think about music they enjoyed before answering five items, including “I love listening to this music” and “Listening to this music is part of who I am”. These items were scored on a 7-point Likert scale, and totals were calculated by summing the scores. The inclusion criteria were set such that participants who scored 20 or above, or averaged 4 or higher for each item, were deemed sufficiently passionate and eligible for inclusion. This is a commonly employed method in studies about passionate fandom [12,48]. Participants were asked a yes/no item to determine whether they identified as a passionate fan of music and were asked to select their favourite genre from a list of 25 different genres, with the option to include their own if it was not on the list of 25.

#### 3.1.3. Procedure

All studies described in this article were approved by the Macquarie University Human Research Ethics Committee (ethics No. 520221112436493). Participants completed an online survey where they were first asked demographic questions including their age, gender, level of music training, and whether they identified as a passionate fan of music. Participants then completed the hedonic and eudaimonic items, the passion items, and the favourite genre question. The study took approximately five minutes to complete, and participants were reimbursed $0.50 AUD for their time as per the guidelines stipulated by the Prolific survey platform.

### 3.2. Results

Factor analysis employing principal axis factoring and a direct oblimin rotation was employed to test the 12 scale items. Sufficient sampling adequacy was verified with the Kaiser–Meyer–Olkin (KMO) statistics, KMO = 0.80, and a significant Bartlett’s test of sphericity, *p* < 0.001. The Kaiser–Guttman rule for extracting factors was employed, whereby only factors with an eigenvalue greater than 1 were deemed significant for factor extraction [49]. Factor analysis identified two factors with eigenvalues greater than 1, which accounted for 54.1% of the variance. The items were loaded according to the constructs in line with the scale they were adapted from, with the six adapted eudaimonic items loading onto one factor and the six adapted hedonic items loading onto another factor. The total explained variance was also higher than in the study the measure was adapted from. There were no issues with cross-factor loadings, as all items loaded uniquely onto their respective factors [50]. Table 1 reports the factor loadings of each item and Table 2 details the descriptive statistics for each item. Internal consistency measures revealed great reliability for the eudaimonic scale (*a* = 0.86) and adequate reliability for the hedonic scale (*a* = 0.77).

The two scales were weakly negatively correlated, (*r* = −0.16, *p* = 0.023). This weak but significant correlation was similar to that observed in the Oliver and Raney (2011) scales used for film [4]. Given that each scale accounts for less than 3% of the other scale, this small negative correlation indicates the two different motivations are predominantly unique rather than bipolar. A paired samples *t*-test revealed that there were also no differences between participants’ mean scores on the hedonic scale (*M* = 5.2, *SD* = 1.0) and the eudaimonic scale (*M* = 5.3, *SD* = 1.0), *t*(201) = 0.565, *p* = 0.573.

At a sample size of 200, items with factor loadings above 0.36 can be deemed significant [51]. All items in the new scale were well above that criterion, except for one item (As long as I am having a good time, I enjoy music that may be considered “basic” or “shallow”). This item was very close to the significance cut-off. While the current factor loadings met Guadagnoli and Velicer’s (1988) guidelines for sufficient and reliable factors, the same analysis was conducted with this item removed to examine whether the results changed notably [46]. While the two factors identified accounted for an increased 3.3% of the variance, the eigenvalues, KMO statistics, and internal consistency coefficient for that subscale all remained very similar. Thus, it was decided that the full 12 items would be retained.

### 3.3. Discussion

Study 1 supported the proposed factor structure for hedonic and eudaimonic motivations for listening to music. Further, fans reported high levels of both hedonic and eudaimonic motivations, with no statistically significant difference between the presence of each. Scores on both subscales were well above the midpoint for each, suggesting that both motivation types are common for a broad range of music fans, as predicted. This finding confirms that passionate music fans strongly appreciate music over and above pleasure and enjoyment. While pleasure remains an integral component of the listening experience, people report an overt drive to engage with music for reflection and to seek out meaningful experiences to a similar extent. What remains unknown is how these different motivations are associated with affective experiences when engaging with one’s favourite music.

## 4. Study 2: Motivations and Affective Outcomes

The first study provided evidence for the existence of two distinct motivation types in music listening: hedonic and eudaimonic motivations. A second study was conducted to further validate the factor structure of this new measure by conducting a confirmatory factor analysis with a new sample of participants, which is best practice for validating new measures [50]. Further, Study 2 sought to understand whether the different motivations for listening to music were associated with different affective responses to music. Such questions will help us establish whether each type of motivation for music listening contributes to the experience of different affective outcomes from the music selected. Specifically, as hedonic motivation suggests an intention to experience pleasure and enjoyment through engagement with media, one’s affective responses to music will likely be overwhelmingly positive. However, eudaimonic motivation suggests an intention to have more complex and challenging experiences that may involve confronting difficult emotions. For this reason, it is predicted that the outcomes will not be as overtly positive and will reflect a more balanced affective response of both positive and negative emotions.

Specifically, it was hypothesised that:

(1) Hedonic motivation for listening to music will be associated with positive affective experiences across all fans.
(2) Eudaimonic motivation for listening to music will be associated with a mixture of positive and negative affective experiences across all fans.

Hedonic motivation for listening to music will be associated with positive affective experiences across all fans.

Eudaimonic motivation for listening to music will be associated with a mixture of positive and negative affective experiences across all fans.

### 4.1. Materials and Method

#### 4.1.1. Participants

The sample consisted of 307 participants (56 males, 247 females, and 4 who identified as neither male nor female or chose not to respond) recruited through the Macquarie University first-year psychology sample. Participants’ mean age was 21.2 (*SD* = 6.8), and ages ranged from 17 to 56. Participants listened to an average of 19.8 h of music per week (*SD* = 15.1). The sample size was deemed appropriate, as it was greater than 300, a commonly cited number in structural equation modelling [41]. This was confirmed through a power analysis calculation, which suggested that 223 participants would be sufficient to detect a small to medium effect size in a model with 2 latent variables and 12 observed variables at a power of 0.8 [52].

#### 4.1.2. Measures

The 12 hedonic and eudaimonic motivation items from Study 1 were presented to participants, as they were all retained by the exploratory factor analysis. The same 5 passion items from the Passion Scale [47] from Study 1 were also presented, with participants again needing to score 20 or above to be eligible for inclusion. Participants were again asked a yes/no item to determine whether they identified as passionate fans of music, and they were asked to select their favourite genre from a list of 25 different genres, with the option to include their own genre if it was not on the list of 25.

The Scale of Positive and Negative Experiences (SPANE) measured participants’ positive and negative affective experiences when listening to their favourite music [53]. The scale consists of two subscales, positive experiences and negative experiences, and both contain six items each. One example of a positive item is “joyful”, and an example of a negative item is “angry”. The SPANE is measured on a 5-point Likert scale, from “Very Rarely or Never” (1) to “Very Often or Always” (5). Scores for each subscale were summed, with each person having a positive and negative subscale score ranging between 6 and 30. The ‘minimum index’ analytical approach was employed to measure mixed emotions but was expanded to include the full range of positive and negative affect measures presented in the study. For example, if a person scored 20 on the summed positive subscale and 15 on the summed negative subscale, their mixed affect score would be 15, as it indicates the extent to which both positive and negative experiences were present. This approach has been used previously to measure mixed affective responses to music [54].

#### 4.1.3. Procedure and Analysis Plan

Participants completed an online survey (administered through Qualtrics) about their experiences when listening to music. Demographic questions were presented, along with the hedonic and eudaimonic motivation items. Participants were then told to think about a piece of music that they “love and enjoy, identify with, strongly value, and spend a lot of time listening to”. They were instructed to listen to this piece of music on their device and then return to the survey, where they filled out the name of the song and provided a link to the song. Participants were then instructed to complete the SPANE and Passion Scale items about the piece of music they just heard. The study took approximately 15 min to complete, and participants were compensated with course credit.

Before hypothesis testing was conducted, confirmatory factor analysis was conducted to further validate the factor structure of the adapted scale. This was conducted using SPSS AMOS 27. Hypothesis testing involved multiple regression analyses conducted using SPSS 27. Outliers were determined as any raw values with *z*-scores greater than ±3.29 standard deviations from the mean and were corrected by assigning new raw values that were either one unit larger or smaller than the next largest or smallest in the data set [45]. Although there were no issues with linearity, homoscedasticity, or multicollinearity, the probability-probability plots and histograms of the residuals revealed that the mixed emotion measure residuals were not normally distributed. Bias-corrected bootstrapping using 1000 samples was conducted for this variable due to it being robust to violations of normality [49]. The bootstrapped regression results did not differ significantly from the original regression results, and so the original regression results were reported and interpreted. The results of the bootstrapped analysis are presented in Appendix A, Table A1.

### 4.2. Results

#### 4.2.1. Confirmatory Factor Analysis

Confirmatory factor analysis (CFA) was conducted with the model parameters estimated based on the model described in Study 1 and using the maximum likelihood function. The model fit was marginally acceptable, *χ*^2^(53) = 171.5, *p* < 0.001, *χ*^2^/df = 3.24, CFI = 0.92, TLI = 0.90, RMSEA = 0.085, 90% CI [0.071, 0.100], but fit far better than a one-factor model (*χ*^2^(54) = 597.1, *p* < 0.001, χ^2^/df = 10.72, CFI = 0.63, TLI = 0.54, RMSEA = 0.178, 90% CI [0.165, 0.192]). Item-factor loadings ranged from 0.43 to 0.81. Internal consistency measures again revealed great reliability for the eudaimonic scale (*a* = 0.88) and sufficient reliability for the hedonic scale (*a* = 0.76). The correlation between the two latent constructs was not significant in this model, *r* = 0.04, *p* > 0.05. Participants again reported high levels of both hedonic motivation (*M* = 5.3, *SD* = 0.9) and eudaimonic motivation (*M* = 5.2, *SD* = 1.1) for listening to music, with no significant difference between the two scores, *t*(306) = 1.67, *p* = 0.097. The findings from the CFA provide further support for the factor structure of the measure with marginal acceptability. Descriptive statistics for each eudaimonic and hedonic item are presented in Table 3, along with the descriptive statistics for the positive and mixed affect scores.

#### 4.2.2. Hypothesis Testing

To test both hypotheses, regression analyses were conducted with the two motivation types as predictors and positive and mixed affective experiences as outcome variables in separate analyses. Results supported H1, revealing that hedonic motivation for listening to music positively predicted positive affective experiences with music across all fans, *β* = 0.26, *t*(304) = 4.62, *p* < 0.001. Eudaimonic motivation was not associated with positive affective experiences, as expected, *β* = −0.04, *t*(304) = −0.65, *p* = 0.518.

Furthermore, H2 was also supported: eudaimonic motivation for listening to music did positively predict mixed affective experiences with music, *β* = 0.12, *t*(304) = 2.14, *p* = 0.033. Finally, hedonic motivation was found to be a negative predictor of mixed affective experiences, *β* = −0.14, *t*(304) = −2.50, *p* = 0.013.

While not hypothesised, eudaimonic motivation for listening to music also positively predicted negative affective experiences with music, *β* = 0.13, *t*(304) = 2.28, *p* = 0.023. Hedonic motivation negatively predicted negative affective experiences, *β* = −0.15, *t*(304) = −2.64, *p* = 0.009.

### 4.3. Discussion

Study 2 provided further evidence for the factor structure of the HEMM scale, with marginally acceptable fit indices. Passionate music fans reported high levels of both motivations, similar to Study 1. Further, a greater presence of hedonic motivation was associated with greater positive affective responses to fans’ preferred music, while a greater presence of eudaimonic motivation was associated with greater mixed affective responses. These findings reveal that fans’ motivations for listening to music generally match the experienced affective outcomes, whereas those motivated by entertainment and pleasure are likely to report positive experiences. Fans reporting wanting to be challenged by music and reflect upon complex matters such as meaning in life are likely to experience a greater balance of positive and negative affect.

Fans of certain genres containing violent themes (e.g., fans of violently themed extreme metal and rap music) tend to experience a greater magnitude of mixed affective responses to their preferred music than fans of non-violent genres [11] and report wanting to feel or confront anger and tension through their listening experience [2]. One prediction is that these fans may be more eudaimonically motivated, causing them to be more driven to listen to music that depicts challenging experiences and reflects difficult themes. Study 3 was designed to test the prediction that mixed affective responses are characteristic of fans of violently themed music due, in part, to having higher eudaimonic motivation compared to fans of non-violent music.

## 5. Study 3: Motivations in Fans of Violently Themed Music

Study 3 was conducted to compare the motivations for music listening between fans of violently themed and non-violently themed music and to further test the reliability and validity of the HEMM scale. Participants were recruited from outside the first-year psychology student sample, increasing the age range of participants and allowing us to achieve a more even gender distribution in our sample. The first hypothesis was, as observed in previous research, that fans of violently themed music would report lower positive affective experiences with their preferred music and higher mixed affective experiences than fans of non-violently themed music (H1). We then predicted that fans of music containing violent themes would report greater eudaimonic motivation for their preferred music than fans of non-violent music (H2). Such an emphasis on eudaimonic motivation would help explain why fans of music containing violent themes experience mixed emotional responses to a significantly greater degree than fans of music without violent themes.

### 5.1. Materials and Method

#### 5.1.1. Participants

Two hundred and thirty-five participants (113 males, 114 females, and 8 who identified as neither male nor female or chose not to respond) were recruited through Prolific. Of these, 121 self-identified as fans of violently themed music and 114 identified as fans of music, but not fans of violently themed music. The mean age of participants was 26.1 (*SD* = 6.4), with ages ranging from 18 to 56. Participants listened to an average of 25.5 h of music total per week (*SD* = 20.0). The sample size was selected based on the power analysis calculation conducted for the previous study, which detailed that 223 participants would be sufficient for detecting a small to medium effect size at a power of 0.8 for this model, as it was the same model as that in Study 2 [52].

Between the two fan groups, there were no statistically significant differences between their level of music training or their hours of total music listened to per week (*p*-values > 0.05). There was a statistically significant difference for hours of violently themed music consumed per week, *t*(233) = 9.01, *p* < 0.001, 95% CI [8.65, 13.50], confirming fans of violently themed music did indeed consume more violently themed music each week (*M* = 13.9 h, *SD* = 12.2) than fans of non-violently themed music (*M* = 2.9 h, *SD* = 5.0). There was also a statistically significant difference between the ages of the two samples, with fans of violently themed music being older (*M* = 27.2 years of age, *SD* = 6.8) than fans of non-violently themed music (*M* = 24.8 years of age, *SD* = 5.7), *t*(233) = 3.00, *p* = 0.003, 95% CI [0.85, 4.09]. As age was not correlated with any of the variables of interest, it was not controlled as a covariate in subsequent analyses. Further details of the demographic information for both groups are presented in Table 4.

#### 5.1.2. Procedure and Analysis Plan

Both groups completed almost identical surveys based on the survey completed in Study 2. The only difference from Study 2 was that participants were asked if they identified as fans of violently themed music, which was defined as music that contains lyrical themes depicting overt acts of violence, as well as were asked how many hours per week they listened to music containing violent themes. Fans of violently themed music were asked to listen to a piece of music that they “love and enjoy, identify with, strongly value, and spend a lot of time listening to”, but were also specifically asked to listen to a song containing violent themes. Otherwise, the order of questions and scales used in Study 3 was the same as in Study 2.

As in Study 2, confirmatory factor analysis using SPSS AMOS 27 was conducted to further validate the adapted scale in this new sample. Hypothesis testing involved independent samples *t*-tests to compare between-group ratings of hedonic and eudaimonic motivations. Outliers were managed in the same way as outlined in the previous studies.

### 5.2. Results

#### 5.2.1. Confirmatory Factor Analysis

CFA was conducted in the same manner as in Study 2, revealing an acceptable model fit, *χ*^2^(53) = 139.0, *p* < 0.001, *χ*^2^/df = 2.62, CFI = 0.92, TLI = 0.92, RMSEA = 0.083, 90% CI [0.067, 0.100]. Item-factor loadings ranged from 0.50 to 0.85 and internal consistency measures revealed great reliability for the eudaimonic scale (*a* = 0.89) and sufficient reliability for the hedonic scale (*a* = 0.77). As in Study 2, there was no significant correlation between the two latent constructs in this model, *r* = −0.02, *p* > 0.05.

#### 5.2.2. Descriptive Statistics and Within-Group Analysis

When all fans were combined, there were no differences between the presence and magnitude of the two motivation types, with participants reporting similarly high levels of hedonic (*M* = 5.5, *SD* = 0.9) and eudaimonic motivations (*M* = 5.4, *SD* = 1.0), *t*(234) = 1.42, *p* = 0.157. However, when divided into two different fan groups, there were significant differences in the magnitude of each type of motivation within each group. Fans of violently themed music reported marginally higher eudaimonic motivation (*M* = 5.6, *SD* = 1.0) than hedonic motivation (*M* = 5.4, *SD* = 1.0), *t*(120) = 1.92, *p* = 0.057. Fans of non-violently themed music reported the opposite, exhibiting significantly higher levels of hedonic motivation (*M* = 5.6, *SD* = 0.8) than eudaimonic motivation (*M* = 5.1, *SD* = 1.0), *t*(113) = 4.11, *p* < 0.001. Means and standard deviations for each eudaimonic and hedonic item and the positive and mixed affect scores for each group are presented in Table 5.

#### 5.2.3. Hypothesis Testing

Fans of violently themed music reported greater mixed affective responses to their preferred music (*M* = 12.0, *SD* = 4.8) than fans of non-violently themed music (*M* = 10.0, *SD* = 3.8), *t*(233) = 3.64, *p* < 0.001. In previous studies, it had been observed that fans of violently themed music reported a more balanced mix of positive and negative affective responses than fans of classical music, with the disparity between positive and negative responses being significantly smaller for fans of music containing violent themes [11]. Similar results were observed in the present study, with fans of violently themed music having lower positive affective responses (*M* = 23.9, *SD* = 3.9) than fans of non-violently themed music (*M* = 25.4, *SD* = 4.0), *t*(233) = 2.85, *p* = 0.005, and higher negative affective responses (*M* = 12.5, *SD* = 5.5) than fans of non-violently themed music (*M* = 10.3, *SD* = 4.4), *t*(233) = 3.40, *p* < 0.001.

Fans of violently themed music also exhibited significantly higher eudaimonic motivation (*M* = 5.6, *SD* = 1.0) than fans of non-violently themed music (*M* = 5.1, *SD* = 1.0), *t*(233) = 3.67, *p* < 0.001. While not specifically hypothesised, there were also significant differences regarding levels of hedonic motivation, such that fans of violently themed music exhibited significantly less hedonic motivation (*M* = 5.4, *SD* = 1.0) than fans of non-violently themed music, (*M* = 5.6, *SD* = 0.8), *t*(233) = 2.26, *p* = 0.025. Hence, both hypotheses were supported.

### 5.3. Discussion

Study 3 further validated the factor structure of the new scale, using a sample outside of the first-year psychology student sample. Such findings detail the scale’s validity in another sample with a wider age range, a more even gender distribution, and participants from outside Australia. Further, the study highlighted differences between the two types of music fans in terms of the presence and magnitude of each type of motivation and their affective responses to music. Fans of violently themed music reported significantly higher levels of eudaimonic motivation and significantly lower levels of hedonic motivation than fans of non-violently themed music, and reported significantly greater mixed affective responses to their preferred music.

These findings suggest that not only do fans of violently themed music engage with such music for non-hedonic purposes such as reflection and meaning making, but they do so to a greater extent than fans of non-violently themed music. This complements previous qualitative reports from fans of violently themed music, who report using such music to highlight, explore, and work through difficult feelings and experiences [26,55]. Although these qualitative reports provided the rationale for hypotheses in Study 3, the ability for people to experience meaning making through overtly violent content has nevertheless been challenged in other media research. Bartsch et al. (2016) found that exaggerated portrayals of blood and gore, implausible plot elements, and overly aestheticized depictions of violence in visual media interfered with meaning making and reflection processes [56]. Further, as music alone lacks a visual narrative, it was uncertain whether violently themed music would provide an environment in which eudaimonic gratifications may be sought. The present study reveals that eudaimonic gratifications are sought to a relatively high extent by many violently themed music fans. As Study 2 detailed the relationship between mixed affective experiences and eudaimonic motivation, the finding in Study 3 that fans of violently themed music report greater eudaimonic motivation and mixed affective experiences supports the tenet of the Uses and Gratifications Theory and the alignment between gratifications sought and gratifications obtained.

Fans of violently themed music also report lower hedonic motivation than fans of non-violently themed genres. Though not formally hypothesised, this is not overly surprising because some items in the scale mention either enjoying music that is simple or basic, or music that is happy and positive. Fans of genres such as death metal commonly report enjoying the technical proficiency displayed in death metal songs [51]. Further, the lyrical content of such music does not often contain themes of happiness, which likely contributes to this finding. Finally, the results detail a better model fit for the measure than in Study 2 with similar trends to those observed in the first two studies, supporting the reliability of the measure.

## 6. General Discussion

Three studies were conducted to develop, validate, and utilise a new measure of hedonic and eudaimonic motivations for listening to music. The first aim was to develop a means of understanding the extent to which music fans listen to music for pleasure (hedonic motivation) and/or to be challenged, provoke thought, and seek meaningful experiences (eudaimonic motivation). The second aim was to test the utility of the new scale in predicting positive and mixed affective responses to music, both in fans of music in general and fans of music with violent themes.

Study 1 was conducted to validate a new measure of motivations for music, adapted from a similar measure created for film viewing motivations [4]. The findings clarified that fans of music across a range of genres have similarly high levels of both types of motivations for listening to music. Study 2 further validated the measures, again showing that fans had similarly high levels of both motivations, as well as revealing that hedonic motivation is associated with positive affective experiences and eudaimonic motivation is associated with mixed affective experiences. Study 3 investigated fans of two different types of music, fans of music containing violent themes and fans of non-violently themed music, and compared the presence of each type of motivation and the nature of their affective responses. Fans of violently themed music reported having higher eudaimonic motivation and lower hedonic motivation for listening to music than fans of non-violently themed music, as well as having a greater presence of mixed affective responses and lower positive affective responses.

Mean ratings for both types of motivation were above five out of seven in all three studies, Together, the studies revealed that passionate music fans are generally highly motivated to listen to music both for pleasure and entertainment purposes as well as to be challenged, reflect, and seek meaning in life. The two types of motivation were also either not correlated or weakly negatively correlated in the three studies, supporting the notion that the two constructs exist as distinct types of motivations rather than opposing ends of a continuum [57]. However, when groups were divided based on whether they exhibited passionate fandom for music containing violent themes or not, differences in the presence of each motivation were evident. Fans of violently themed music were more highly motivated by music that was challenging and made them reflective about themselves and the world and less motivated by entertainment and pleasurable experiences when compared to fans of non-violent music.

The present study supported the link between the two different motivations and two different affective responses to music across a range of fans. This finding supports a key tenet of the Uses and Gratifications Theory and previous research findings that suggest that people actively select music or other media that will gratify their cognitive and affective needs, such as fans who actively pursue enjoyment and pleasure through their favourite music largely experiencing positive affective outcomes [24,58]. Eudaimonically motivated fans experienced a greater mix of positive and negative affect. As eudaimonic motivations have been described to reflect complex, higher-order needs such as insight into the human condition [59], it would not be expected that being motivated to address such complex needs would lead to purely positive experiences. Rather, pursuing such complex needs is likely to lead to a breadth of affective experiences, instead of simply enjoyment or happiness [4]. The present study suggests that seeking to address complex needs through music can lead to the co-activation of positive and negative affect, such as feeling happy, joyful, sad, and afraid, in response to one’s favourite music across passionate fans of a broad range of music genres.

While fans of a broad range of different genres seek and obtain these eudaimonic experiences with music, fans of music containing violent themes do this to a greater extent. Understanding the motivations that drive people to engage with violently themed music was a key motivator for the present research. Previous lines of research have largely neglected to understand the specific motivations that fans may have for wanting to continually engage with violently themed music, instead largely focusing on assessing preferences for these genres and potential correlations with problem behaviours (for reviews, see [60,61]). Qualitative reports suggest that fans listen to extreme metal music such as death metal to elucidate their current feelings and stresses, work through difficult emotions, and understand more about the difficult aspects of life, as well as for sonic motivations such as the virtuosic talents of the musical performers [26,55]. The present study provides a validated tool for systematically assessing fan motivations and shows that, while pleasure remains a key motivator of the listening experience, the overtly violent context that the music provides can facilitate self-reflection and provide an opportunity for fans to gain insights about themselves and the world.

Fans of violently themed music reported predominantly positive affective experiences in response to their self-selected violently themed music but did experience these to a lesser extent than fans of non-violently themed music. Further, fans of violently themed music reported a greater mixed affective response to their self-selected music than fans of non-violently themed music. These findings support previous reports of mixed emotional responses by fans of violently themed music and extend this knowledge by using the validated minimum index measure and a comparison group comprising a broader sample of fans of non-violently themed music [11]. These mixed affective experiences appear to be deliberately pursued by fans, with the content of the music providing fans with a sense that more eudaimonic experiences can be sought and obtained.

We propose that being motivated to be reflective and pursue value and meaning from musical engagement can be an adaptive process for all music fans, even though the immediate affective outcomes may not always be directly positive. By being able to learn about challenging experiences, engage in self-reflection, and pursue meaning through music, fans can gain insight into the difficult aspects of the human experience. Fans may be able to use this process to further develop their sense of self, their understanding of their place in the world, and understand more about their struggles and challenges. It has been theorised that adaptive music listening does not necessarily mean avoiding difficult stimuli and that engaging with music containing difficult themes can promote well-being [30]. Ryff and Keyes’ (1995) conceptualisation of psychological well-being involves six dimensions, three of which are aspects of eudaimonic well-being: personal growth, purpose in life, and self-acceptance [62]. Previous research has also detailed that possessing eudaimonic and hedonic motivations for other non-music activities significantly contributes to psychological well-being in both distinct and complementary ways [63]. Therefore, having both motivations for engagement with music may facilitate positive affective experiences with music, as well as other, more complex eudaimonic experiences that also foster and facilitate adaptive outcomes and well-being. This can even be the case for fans of overtly violent content, where fandom in such genres has been shown to positively contribute to psychological well-being [12].

Future investigations should seek to further understand how mixed affective experiences from eudaimonically motivated engagement with music can facilitate adaptive outcomes such as psychological well-being. While the adaptive outcomes from possessing eudaimonic motivation are theorised to be through processes such as self-reflection and personal growth, it is important to systematically investigate this in the future. Further, understanding a way of measuring the affective responses associated with these self-reflective and personal growth processes will assist in comprehensively understanding the experiential process of music engagement, from motivation to general emotional and experiential outcomes.

The three studies provide an important and novel validated measure to understand and measure hedonic and eudaimonic motivations for music. Future research should continue to investigate this measure while addressing the limitations of this investigation. Firstly, our study did not investigate specific fan populations outside of the distinctions between violently themed and non-violently themed music. Secondly, as participants completed the study online, the music listening environment was not controlled or necessarily consistent across participants. As the outcomes of listening experience strongly depend on personal and social contexts [8,64], future research may seek to conduct research with more controlled listening environments. In Study 2, participants were all university psychology students and predominantly women, while Study 3 relied on participants from a survey platform. These samples are biased towards more educated and digitally active participants. Conducting surveys with fans in non-online spaces, for example, surveying people at a live music event, would help to further validate the measure beyond digitally active participants. Future research could focus on the relationship between preferences for violently themed music and education levels or cognitive styles, as preferences for heavy metal and other intense genres have been associated with ‘systemisers’, a cognitive style associated with interest and ability in analysing how systems work [65]. However, to our knowledge, no research has investigated such questions specifically in the context of violence and across different genres containing violent themes.

Further, only basic emotions were assessed, and the experience of more complex emotions such as being moved, inspired, or nostalgic were not captured. Participants self-selected their favourite music, so they underwent a vast range of evaluative and appraisal processes when engaging with this music, which is likely to have led to many complex emotional experiences (for a discussion, see [66]). Future research should seek to understand how different motivations may be associated with more complex emotional experiences with music, with the expectation that eudaimonic motivation will be associated with more complex emotional experiences. In addition, the measure of mixed emotions, while a commonly used measure in the field of mixed emotion research, appeared to correlate strongly with the negative emotion scores, as the predominant responses from participants reflected positive affective experiences. In the future, other behavioural mixed emotion measures, such as the frequency of a button-press response that participants make when experiencing two opposing yet co-occurring emotions (see [39]), may be used to measure mixed emotions more accurately.

While it was confirmed that the violently themed fan group did report fandom for violently themed music and did listen to music containing violent themes in the listening section, it is possible that these fans were fans of many different music genres and selected very specific types of music to satisfy different motivations. For example, a fan of extreme metal music may exhibit high levels of both eudaimonic and hedonic motivations but listen to extreme metal to satiate eudaimonic gratifications and another genre, such as rock music, to satiate hedonic motivation (or vice versa). Gathering a more nuanced profile of listeners’ habits is an important avenue for future research. Finally, future research that investigates the connection between motivations for engaging with music and the resultant outcomes should include personality variables that may mediate the experience of desired outcomes, such as depression and anxiety [17], the presence of harmonious or obsessive passion [11], or tendency for healthy or unhealthy music engagement [35].

## 7. Conclusions

By adapting and validating a measure for eudaimonic and hedonic motivation for music, a deeper understanding of listeners’ purposes for listening to music can be obtained. This scale highlights that listeners intend to listen to music for both pleasure and more meaningful and challenging purposes, and these are reflected in the affective experiences that fans have during the listening experience. Noting the limitations of this study, the measure can be applied in the future to understand more about the motivations of listeners of different styles of music, as well as to understand how these motivations can impact broader and more complex aspects of fans’ musical appreciation.

## Figures and Tables

**Table 1:** Factor loadings for each scale item in Study 1.

Items	Eudaimonic Motivation	Hedonic Motivation
I like music that challenges my way of seeing the world	0.603	0.025
My favourite songs are the ones that make me think	0.752	−0.031
I like music that makes me more reflective	0.793	−0.046
I like music that focuses on meaningful human conditions	0.654	−0.016
I am moved by music about the search for meaning in life	0.714	0.018
I like music that has profound messages to convey	0.730	0.017
It’s important to me that I have fun when listening to music	0.166	0.715
My favourite music is happy and positive	−0.087	0.519
I most enjoy music that entertains me	0.034	0.800
I find that even simple music can be enjoyable as long as it is fun	0.081	0.645
The most important purpose of music is to be entertaining	−0.119	0.634
As long as I am having a good time, I enjoy music that may be considered “basic” or “shallow”	−0.069	0.383

**Table 2:** Descriptive statistics for each item in Study 1.

Construct	Items	*M*	*SD*
Eudaimonic Motivation			
	I like music that challenges my way of seeing the world	5.40	1.16
	My favourite songs are the ones that make me think	5.23	1.36
	I like music that makes me more reflective	5.46	1.14
	I like music that focuses on meaningful human conditions	4.98	1.31
	I am moved by music about the search for meaning in life	5.09	1.34
	I like music that has profound messages to convey	5.54	1.13
Hedonic Motivation			
	It’s important to me that I have fun when listening to music	5.90	1.26
	My favourite music is happy and positive	4.37	1.54
	I most enjoy music that entertains me	5.50	1.24
	I find that even simple music can be enjoyable as long as it is fun	5.63	1.19
	The most important purpose of music is to be entertaining	4.57	1.68
	As long as I am having a good time, I enjoy music that may be considered “basic” or “shallow”	5.39	1.43

**Table 3:** Descriptive statistics for each item in Study 2.

Construct	Items	*M*	*SD*
Eudaimonic Motivation			
	I like music that challenges my way of seeing the world	5.06	1.33
	My favourite songs are the ones that make me think	4.96	1.45
	I like music that makes me more reflective	5.29	1.32
	I like music that focuses on meaningful human conditions	5.17	1.39
	I am moved by music about the search for meaning in life	4.99	1.34
	I like music that has profound messages to convey	5.47	1.13
Hedonic Motivation			
	It’s important to me that I have fun when listening to music	5.98	1.12
	My favourite music is happy and positive	4.41	1.52
	I most enjoy music that entertains me	5.74	1.09
	I find that even simple music can be enjoyable as long as it is fun	5.65	1.15
	The most important purpose of music is to be entertaining	4.58	1.60
	As long as I am having a good time, I enjoy music that may be considered “basic” or “shallow”	5.36	1.30
Affective Experiences			
	Positive affective responses	25.41	3.83
	Negative affective responses	11.21	4.74
	Mixed affective responses	11.02	4.37

**Table 4:** Demographics of the two fan groups in Study 3.

			Group Means (*SD*)			
Fan Group	*n*	Gender (M | F | NB/Other/Choose Not to Disclose)	Age	Hours Total Music	Hours Violently Themed Music	Years Music Training
Fans of Violently Themed Music	121	65 | 51 | 5	27.24 (6.79)	26.00 (21.12)	13.93 (12.18)	3.25 (5.54)
Fans of Non-Violently Themed Music	114	48 | 63 | 3	24.77 (5.73)	25.40 (20.11)	2.99 (5.29)	2.91 (5.66)

**Table 5:** Descriptive statistics for each item in Study 3.

		Group Means (*SD*)	
Construct	Items	Fans of Violently Themed Music	Fans of Non-Violently Themed Music
Eudaimonic Motivation			
	I like music that challenges my way of seeing the world	5.82 (1.04)	5.42 (1.10)
	My favourite songs are the ones that make me think	5.53 (1.35)	4.76 (1.51)
	I like music that makes me more reflective	5.64 (1.16)	5.35 (1.21)
	I like music that focuses on meaningful human conditions	5.60 (1.24)	5.09 (1.27)
	I am moved by music about the search for meaning in life	5.36 (1.39)	4.84 (1.44)
	I like music that has profound messages to convey	5.74 (1.20)	5.32 (1.38)
Hedonic Motivation			
	It’s important to me that I have fun when listening to music	6.12 (1.11)	6.35 (0.84)
	My favourite music is happy and positive	4.18 (1.67)	4.72 (1.50)
	I most enjoy music that entertains me	5.85 (1.09)	6.02 (0.96)
	I find that even simple music can be enjoyable as long as it is fun	5.82 (1.08)	5.99 (0.95)
	The most important purpose of music is to be entertaining	5.04 (1.74)	5.08 (1.43)
	As long as I am having a good time, I enjoy music that may be considered “basic” or “shallow”	5.29 (1.58)	5.71 (1.31)
Affective Experiences			
	Positive affective responses	23.89 (3.91)	25.37 (4.02)
	Negative affective responses	12.52 (5.54)	10.30 (4.37)
	Mixed affective responses	12.02 (4.79)	9.97 (3.76)

## Data Availability

The data presented in this study are openly available through OSF at https://osf.io/grbd7/ (accessed on 29 January 2023).

## Appendix A

**Table A1:** Results of the bootstrapped regression analyses that violated assumptions in Study 2.

Construct	*β* Hedonic	*SE* Hedonic	*β* Eudaimonic	*SE* Eudaimonic
Mixed Affect	−0.70 * (−1.24, −0.17)	0.28	0.49 * (0.03, 0.93)	0.23

Note. * *p* ≤ 0.05; *β* = unstandardised beta coefficients from bootstrapped multiple regression, with 95% bias corrected and accelerated confidence intervals presented in brackets. SE = standard errors of the unstandardised beta coefficients.
